# In Vitro Evaluation of Antioxidant Properties of Ten Iranian Medicinal Plants

**DOI:** 10.5812/ircmj.10264

**Published:** 2014-06-05

**Authors:** Mahmoud Rafieian-Kopaei

**Affiliations:** 1 Medical Plants Research Center, Shahrekord University of Medical Sciences, Sharekord, IR Iran

**Keywords:** Antioxidant, Evaluation, Plants, Medicinal

***Dear Editor,***

We read with interest the recently published article in the esteemed Iranian Red Crescent Medical Journal, by Moein et al., entitled “In vitro antioxidant properties evaluation of 10 Iranian medicinal plants by different methods”(1) in which antioxidant properties of ten Iranian medicinal plants were evaluated with two reliable models. The authors found very high levels of phenolic compounds and antioxidant activities in *Verbascum sinuatum* L. and *Rosa damascena* Mill so that the antioxidant activities in terms of 1, 1-diphenyl-2-picrylhydrazyl (DPPH) radical scavenging model in *V. sinuatum* L. (IC_50_ = 263.52 ± 5.981 μg/mL) and *R. damascena* Mill (IC_50_ = 287.9 ± 5.675 μg/mL) were even higher than gallic acid (IC_50_ = 25.32 ± 5.593 μg/mL), which is usually used as a standard antioxidant. The authors have skipped from these valuable results that might have tremendous clinical implications. Most of the untreatable diseases or toxic effects of drugs are related to free radicals and consumption of antioxidants may scavenge these free radicals. It is being increasingly recognized that many of currently untreatable or hard to treat diseases are due to the "oxidative stress". Oxidative stress is initiated by free radicals and results from an imbalance between formation and neutralization of these highly active compounds. Free radicals seek stability through electron pairing with biological macromolecules such as lipids, proteins, and DNA in healthy human cells and cause lipid peroxidation along with protein and DNA damage. These changes highly contribute to the atherosclerosis, cardiovascular diseases, cancer, ageing, and inflammatory diseases (2-5). Human cells protect themselves against free radical damage by antioxidant compounds such as ascorbic acid, tocopherol, and glutathione or enzymes such as catalase and superoxide dismutase (6-8). These protective mechanisms might be disrupted by various pathological processes. In these situations, antioxidant supplements are vital to combat oxidative damage (6-8). Recently, high attention has been directed towards the ethnomedicines with strong antioxidant properties but low toxicities. Although, in Moein et al. article, (1) the extracts were not examined for different reactive oxygen species such as “singlet oxygen, superoxide, hydrogen peroxide, hydroxyl, nitric oxide and peroxynitrite”, total antioxidant activities were examined and showed to be very high for *V. sinuatum* L. and *R. damascena* Mill. Plants with antioxidant activities have shown promising results in various conditions including prevention or treatment of hyperlipidemia (9), diabetes (10), cancer (11), ischemia, and inflammatory diseases (4). These plants have also protective effects against toxicity of different drugs (12-15). Therefore, the plants *V. sinuatum* L. and *R. damascena* Mill, which have high levels of antioxidants, are promising and worth examining for the conditions that cause extensive damage to tissues and biomolecules leading to various disease conditions, especially degenerative diseases and lysis degeneration.
